# Supplementary figures and images for: Contactin-1 links autoimmune neuropathy and membranous glomerulonephritis
Source: PLoS One. 2023 Mar 9;18(3):e0281156. doi: 10.1371/journal.pone.0281156 (PMC9997925; doi:10.1371/journal.pone.0281156)

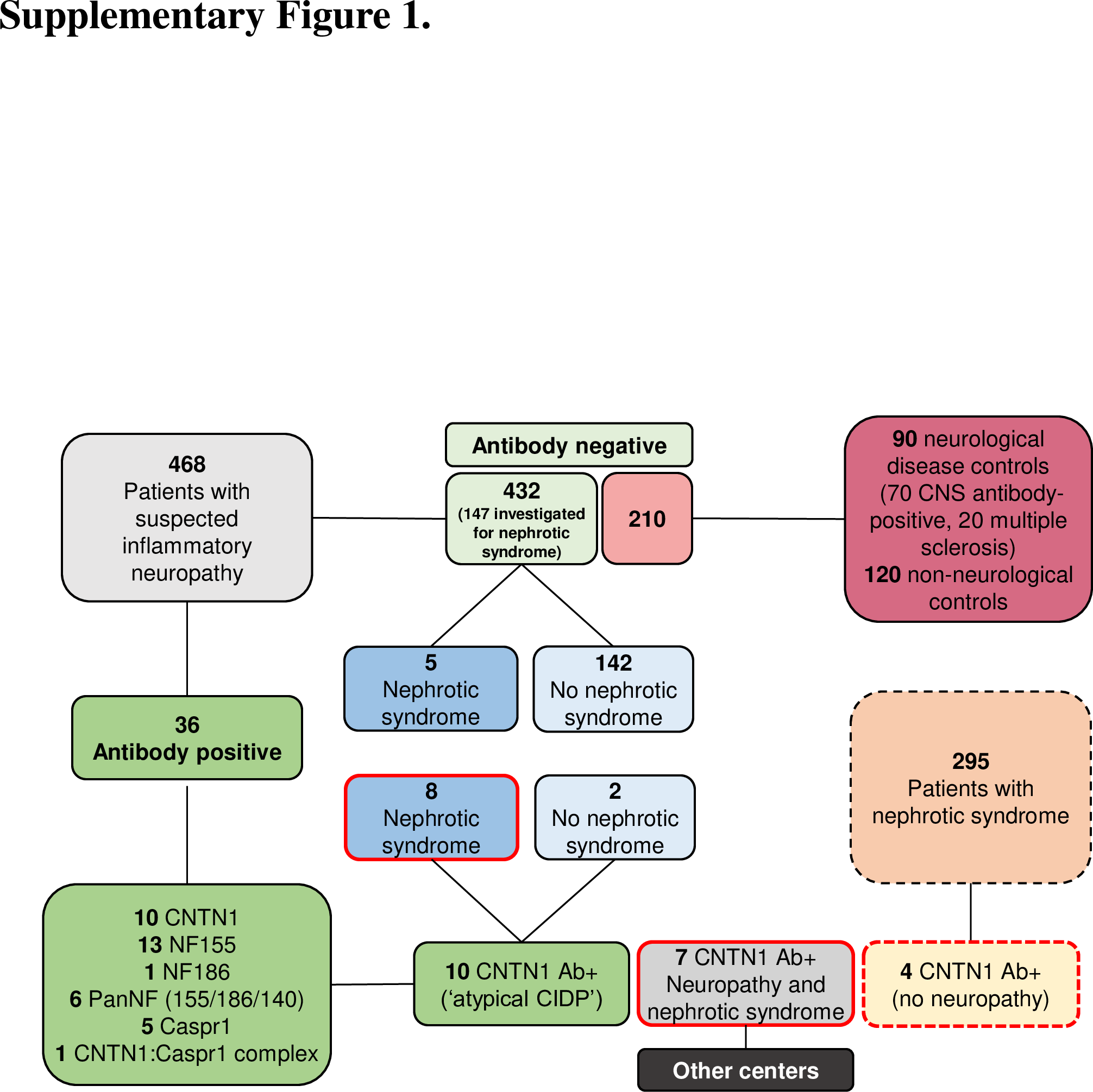

Supplement: S1 Fig — (TIFF) [file pone.0281156.s002.tiff]

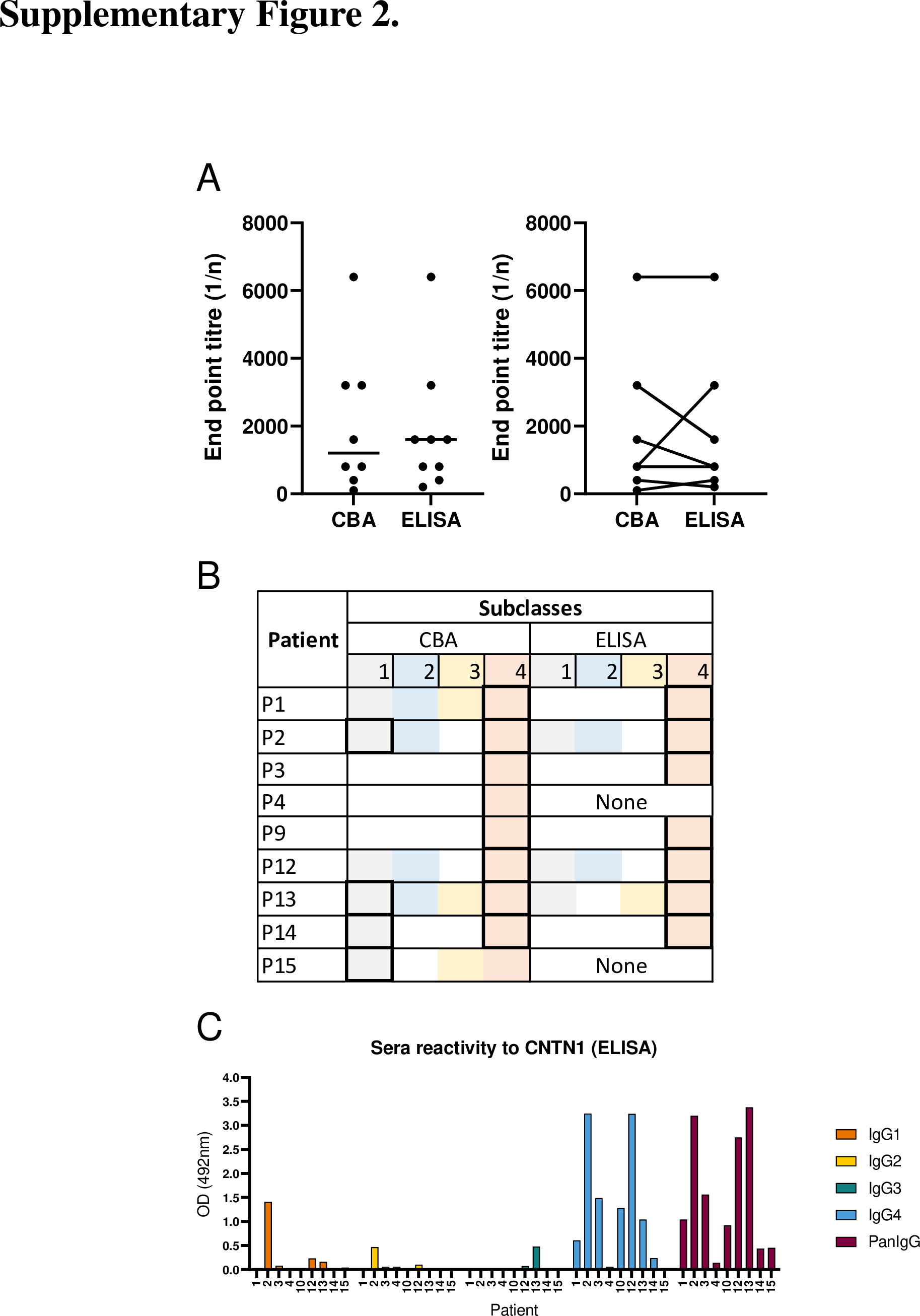

Supplement: S2 Fig — (TIFF) [file pone.0281156.s003.tiff]

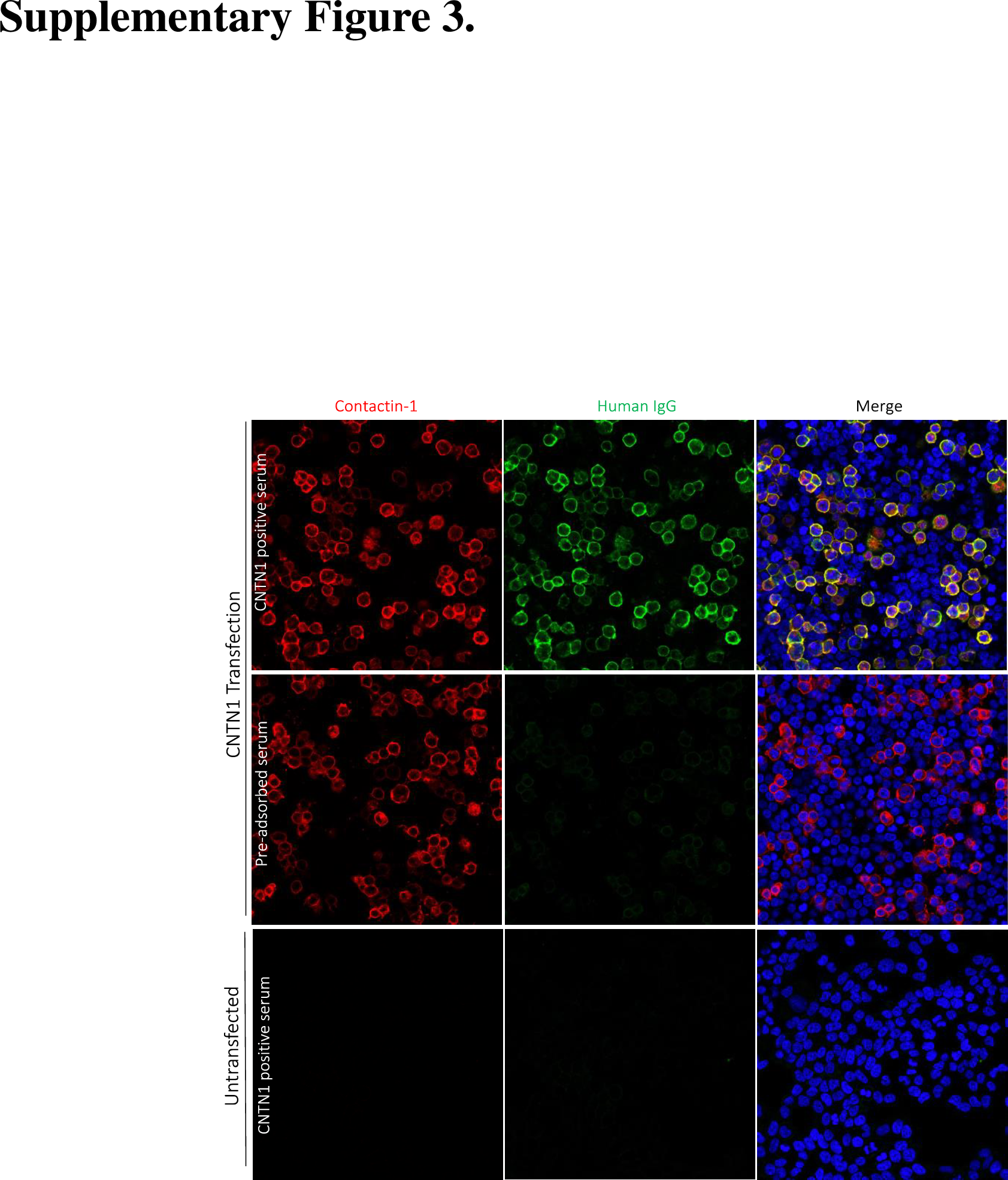

Supplement: S3 Fig — (TIFF) [file pone.0281156.s004.tiff]

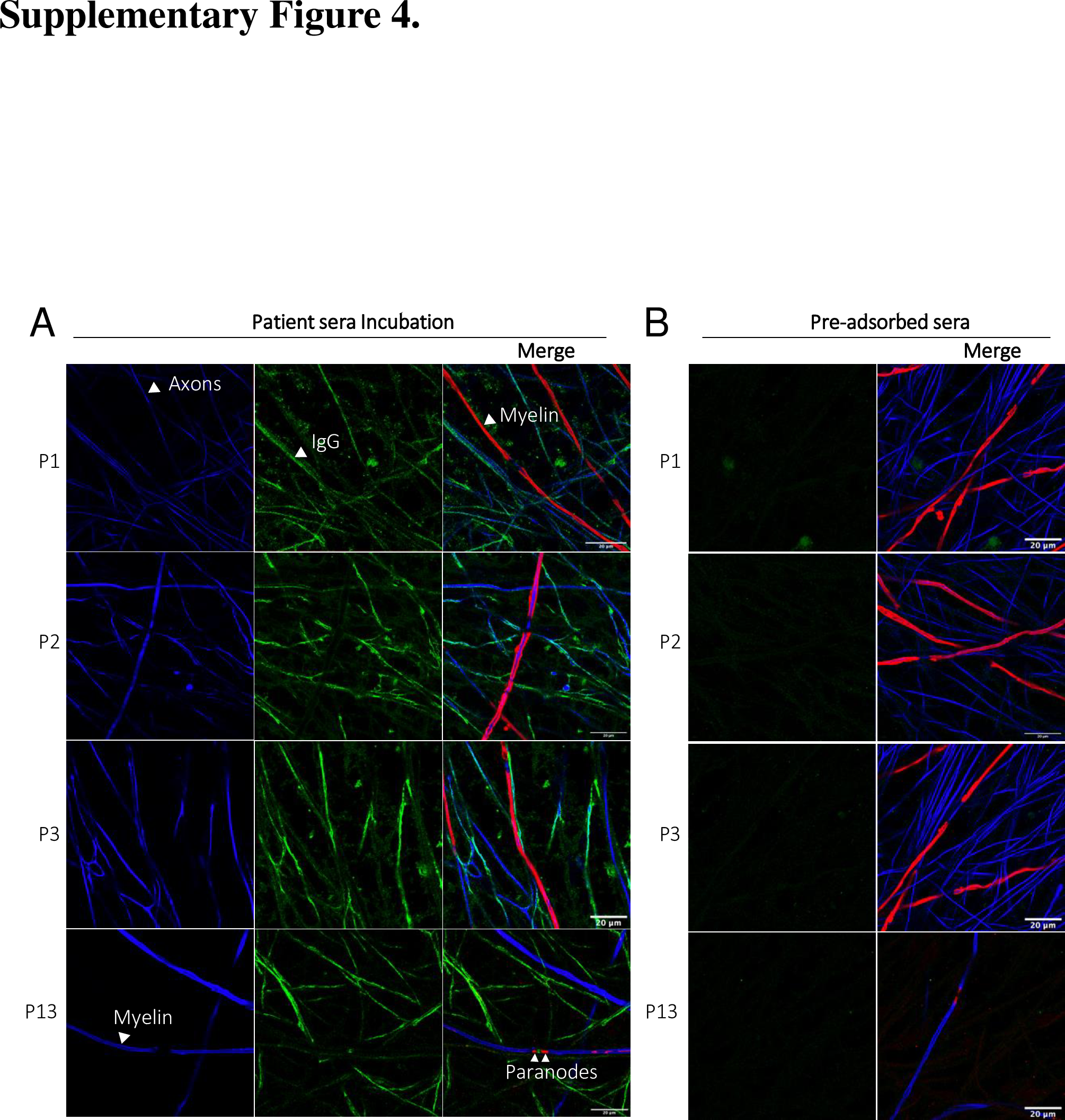

Supplement: S4 Fig — (TIFF) [file pone.0281156.s005.tiff]

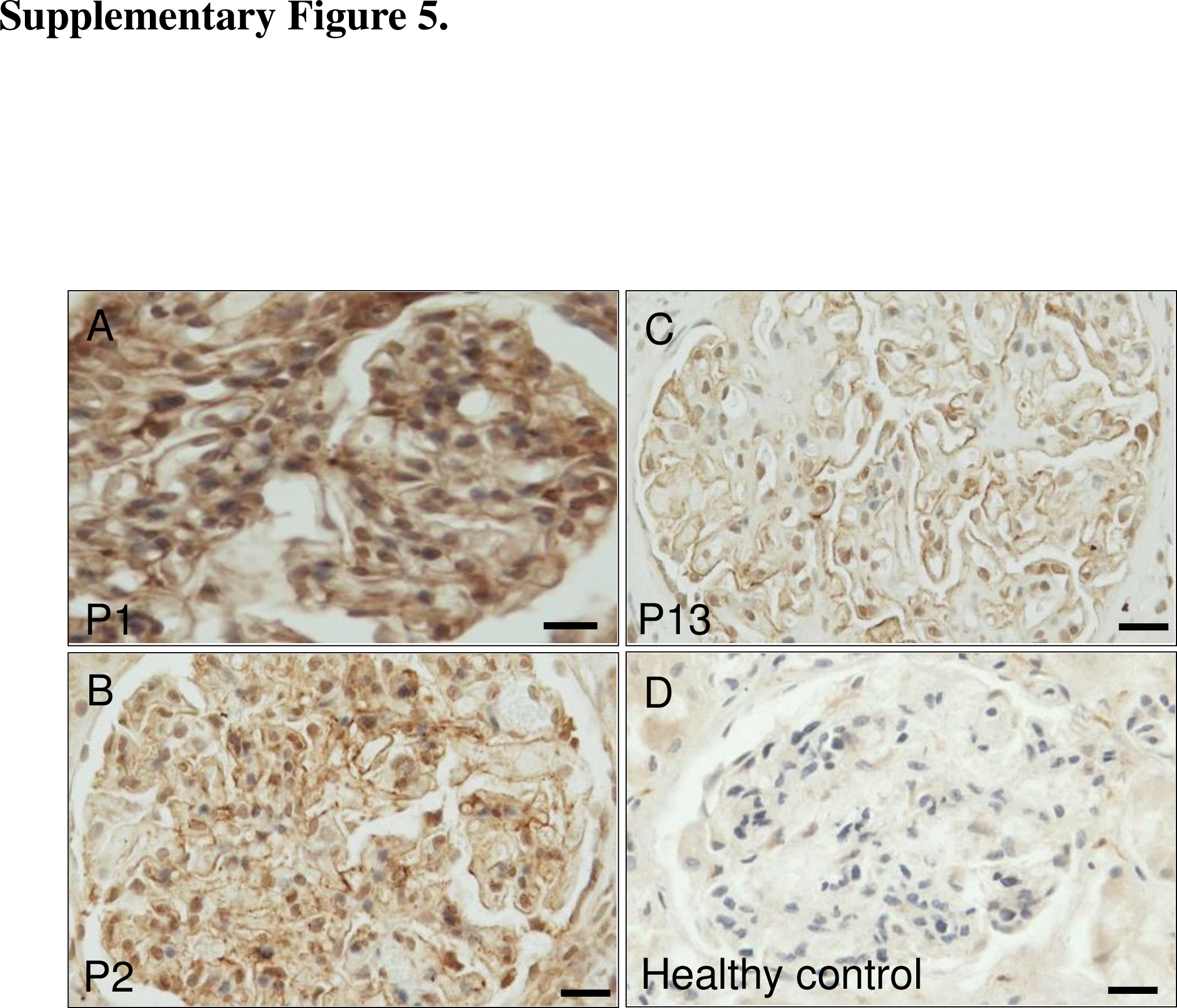

Supplement: S5 Fig — (TIFF) [file pone.0281156.s006.tiff]

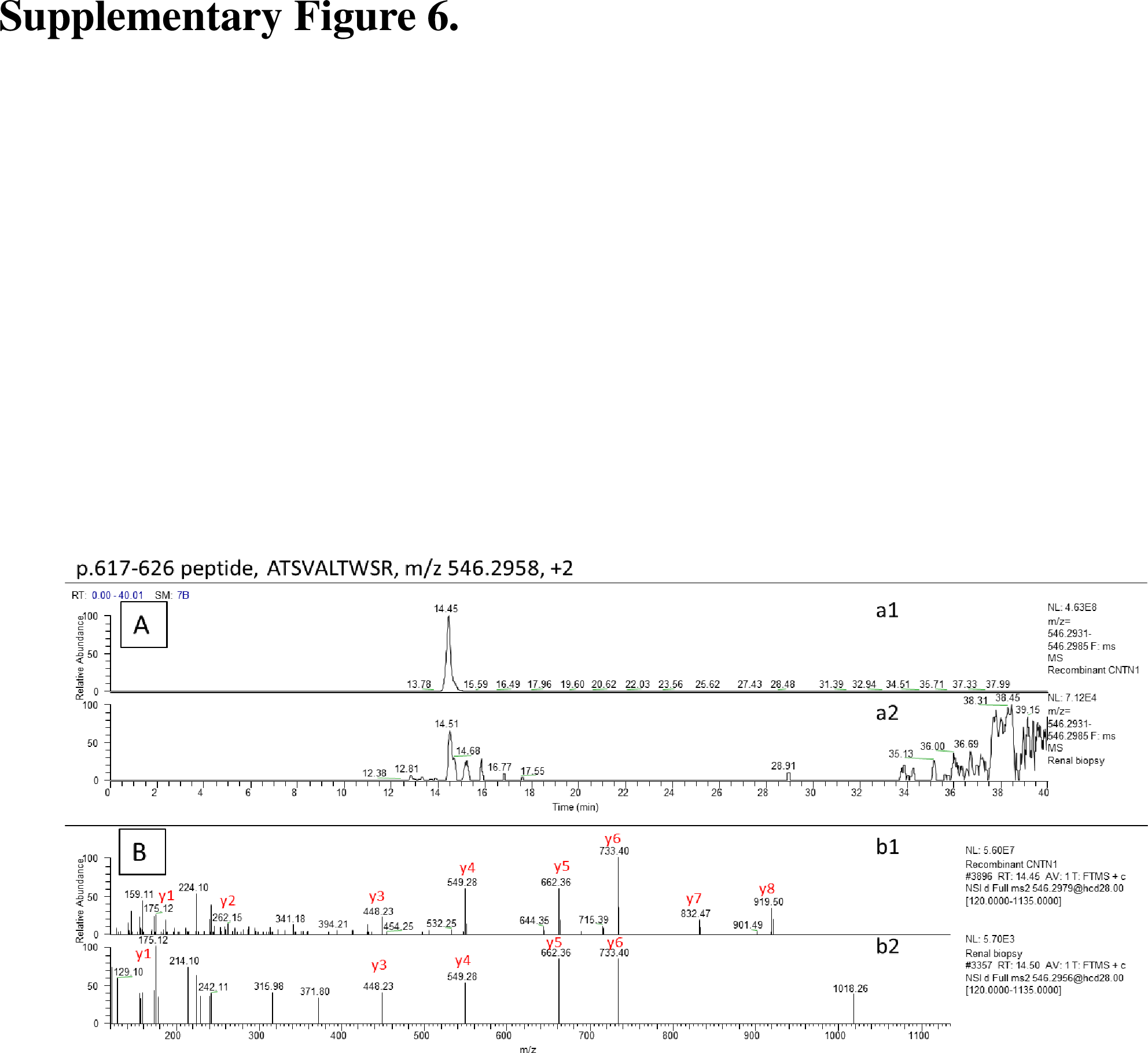

Supplement: S6 Fig — (TIFF) [file pone.0281156.s007.tiff]

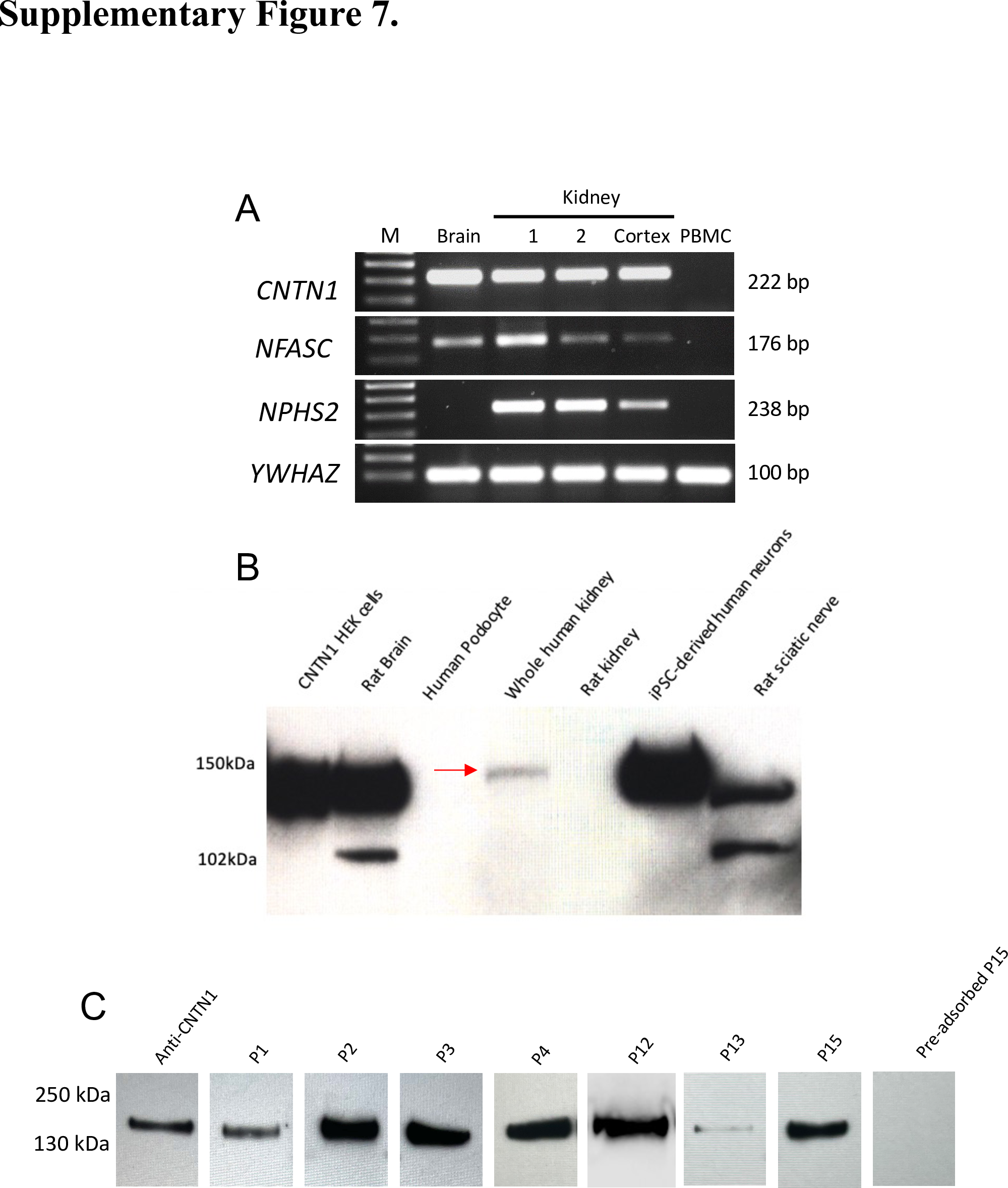

Supplement: S7 Fig — (TIFF) [file pone.0281156.s008.tiff]

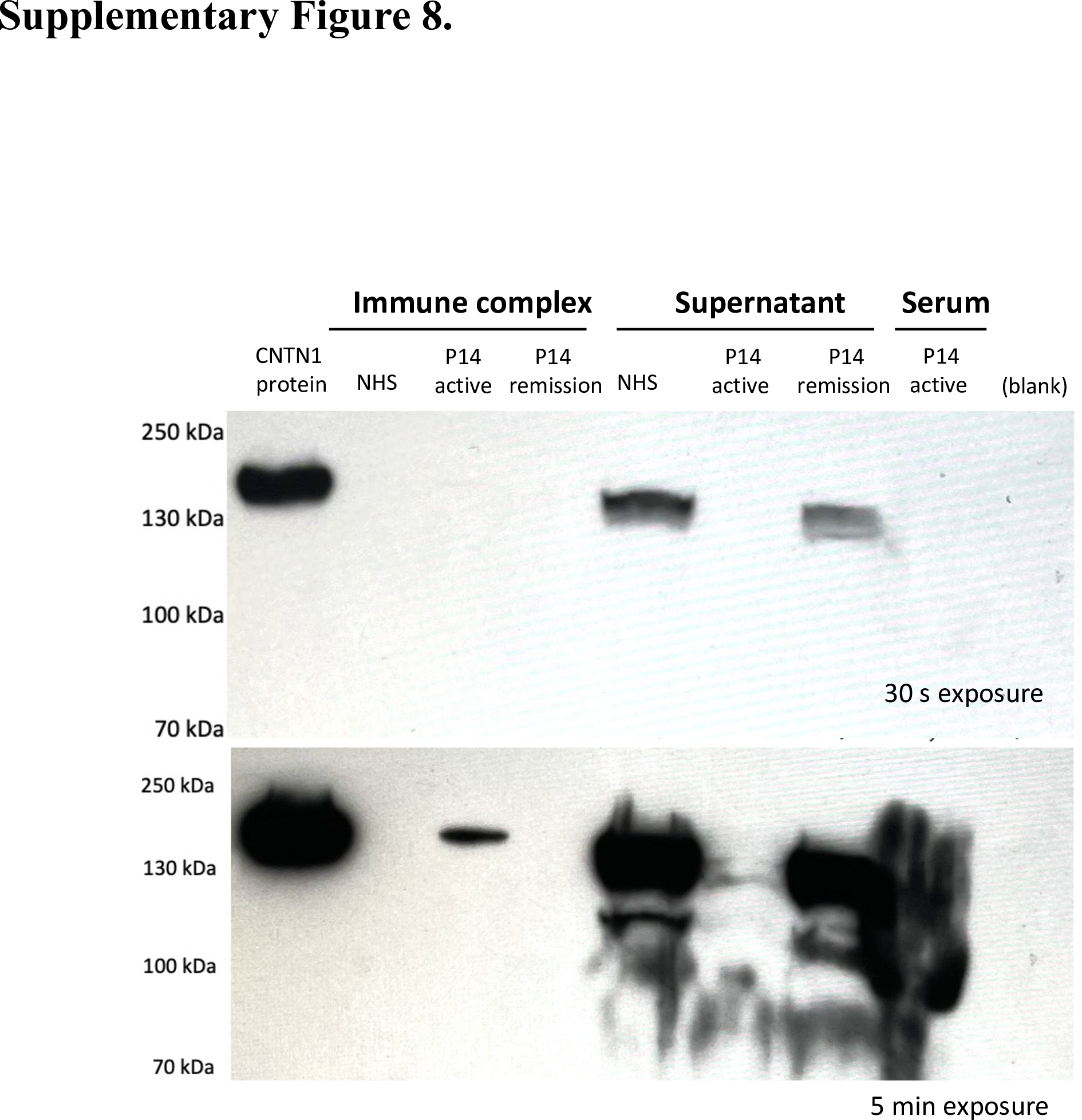

Supplement: S8 Fig — (TIFF) [file pone.0281156.s009.tiff]
