# Supplementary material for: Contactin-1 links autoimmune neuropathy and membranous glomerulonephritis
Source: PLoS One. 2023 Mar 9;18(3):e0281156. doi: 10.1371/journal.pone.0281156 (PMC9997925; doi:10.1371/journal.pone.0281156)
Supplement: S1 File — (DOCX) [file pone.0281156.s010.docx]

Table of Contents

[METHODS AND MATERIALS 2](#_Toc92801978)

[Nodal/paranodal antibody testing and CNTN1 antibody binding to peripheral nerve 2](#_Toc92801979)

[Cell based assay (CBA) 2](#_Toc92801980)

[Enzyme-linked immunosorbent assay (ELISA) 2](#_Toc92801981)

[Myelinating co-cultures 3](#_Toc92801982)

[Mouse sciatic nerve teased fibres 4](#_Toc92801983)

[CNTN1 identification 5](#_Toc92801984)

[Human samples 5](#_Toc92801985)

[Immunohistochemistry 6](#_Toc92801986)

[Western Blot 7](#_Toc92801987)

[Serum CNTN1 protein measurement 9](#_Toc92801988)

[End-point polymerase chain reaction (PCR) 9](#_Toc92801989)

[Laser microdissection and mass-spectrometry proteomic analysis 10](#_Toc92801990)

[FIGURES 12](#_Toc92801991)

[TABLES 20](#_Toc92801992)

[REFERENCES 28](#_Toc92801993)

# **METHODS AND MATERIALS**

## **Nodal/paranodal antibody testing and CNTN1 antibody binding to peripheral nerve**

### Cell based assay (CBA)

Human embryonic kidney 293T (HEK) cells were plated at a density of 75,000 cells/13mm coverslip, and either co-transfected with human DNA plasmid constructs for CNTN1 (EX-A1153-MO2 GeneCopoeia, Maryland, US) and Caspr1 (EX-MO417-MO2 GeneCopoeia) or mono-transfected with NF155 (RC228652, Origene) or NF186 (courtesy of Jerome Devaux, University of Marseille), diluted in Jet-PEI transfection reagent (101-10; Polyplus). Patient sera were initially diluted to 1:100 in DMEM/1% bovine serum albumin (BSA) for incubation with neurofascin transfected live cells, and 1:40 for CNTN1/Caspr1 co-transfected live cells. For pre-adsorption assays CNTN1 positive patient sera was incubated with 1ug of CNTN1 protein (10383-H08H, Sino Biological Inc) overnight at 4°C prior to application to the HEK cells. Co-incubation with commercial primary antibodies chicken anti-neurofascin, (1:1000) (AF3235, R&D systems), mouse anti-Caspr1, (1:1000) (P97846, Neuromab), or goat anti-contactin-1 (1:1000) (AF904, R&D systems) were used to confirm successful transfection and to assess for co-localisation with any bound human IgG. Cell permeabilization with ice-cold methanol for 25 minutes was performed after incubation of sera with live cells and subsequent fixation with 4% PFA, prior to incubation with Caspr1 commercial antibody. Secondary antibodies used were goat anti-human IgG-Fc specific-Alexa Fluor 488 (1:750) (H10120, Life Tech), goat anti-chicken Alexa Fluor 546 (1:1000) (A11040, Life Tech), goat anti-mouse Alexa Fluor 546 (1:1000) (A11003; Life Tech), and unconjugated mouse anti-human IgG subclass 1-4 antibodies (1:100) (I2513, I25635, I7260 I7385; Sigma Life Sciences) which were used with a fluorescently tagged tertiary antibody goat anti-mouse Alexa Fluor 488 (1:750) (A11029, Life Tech).

### Enzyme-linked immunosorbent assay (ELISA)

Individual wells of Nunc Maxisorp ELISA plates (Fisher Scientific) were coated with either human recombinant neurofascin-155 (NF155) (8208-NF, R&D systems), NF186 (TP329070, OriGene Technologies) or CNTN1 (10383-H08H, Sino Biological Inc) as previously described.[1] Sera were initially screened at 1:100 dilution in 5% milk. Anti-human IgG (Fc-specific) peroxidase-conjugated anti-human IgG (A0170, Sigma) was used at 1:3000. The end-point titre of positive samples was assessed by serial doubling dilution from 1:100 to 1:6400, and the IgG subclass was identified using subclass specific, HRP-conjugated secondary antibodies (1:1000) (MH1715, MH1722, MH1732, MH1742; Thermo Fisher Scientific). The detection reaction was performed using o-Phenylenediamine dihydrochloride (OPD, Sigma) and optical densities measured at 492nm. Wells with ODs greater than 0.1 above uncoated control wells were considered positive.

### Myelinating co-cultures

Human fibroblasts reprogrammed to pluripotent stem cells (iPSC) from two healthy control lines (AD2 and AD3) were obtained from the StemBANCC consortium at the University of Oxford. iPSC were differentiated to sensory neurons using a combination of small-molecule mediated dual-SMAD inhibition and WNT activation according to a previous protocol.[2] On day 11 of differentiation, sensory neuron precursors were seeded onto 13 mm diameter glass coverslips (approximately 20,000 cells per coverslip) in 24-well plates. Neurons were fed with N2 neurobasal media supplemented with human recombinant growth factors (ß-NGF, BDNF, GDNF, NT-3) supplemented with Rho-associated, coiled-coil containing protein kinase (ROCK) inhibitor (10 µM) (Tocris, Bio-Techne) on days 11-12, CHIR99021 (3 µM) (Sigma) on days 11-14 and cytosine arabinoside (Ara-C) (1 µM) (Sigma) on days 12-14. Neurons were incubated at 37°C in 5% CO_2_ for 4 weeks with twice-weekly medium changes.

Myelination was achieved by the addition of rat Schwann cells. Animals were killed by rising concentration of CO_2_ in accordance with Schedule 1 of the UK Home Office Animals (Scientific Procedures) Act 1986. Schwann cells were harvested from sciatic nerves of Wistar rat pups (P2-3) by digestion in a mixture of collagenase (3mg/ml) (Worthington, Lorne Labs) and dispase II (3.5mg/mL) (Roche) for 1 h at 37°C with frequent gentle agitation. Schwann cells were then added at 20,000 cells/well in Schwann cell basal media [containing: (CS-FBS) (10%), insulin (5 mg/ml) (Sigma), holo-transferrin (100 mg/mL) (Sigma), rhNGF (25 ng/mL) (Peprotech) (Sigma), Selenium (25 ng/mL) (Sigma), 25 ng/ml thyroxine (Sigma), progesterone (30 ng/ml) (Sigma), triiodothyronine (25 ng/mL) (Sigma) and putrescine 8 mg/mL (Sigma) in DMEM/F12 media (Gibco, Life Technologies)] to establish myelinated co-cultures. Schwann cells align to the axons, and myelination begins after 1 week following incubation with myelination media [containing: 5% CS-FBS, ascorbic acid (25 µg/mL), phenol-free matrigel (1:300) (Corning) and hrNGF (25 ng/mL) in ‘complete’ neurobasal medium], which was changed twice weekly thereafter until mature myelin internodes were observed (around 4 weeks).

Mature myelinated neurons were incubated with patient sera diluted at 1:100 in N2 neurobasal media with 1% BSA and human nerve growth factor (NGF) (25 ng/ml) for 1 h at 37°C. For pre-adsorption assays patient sera was first incubated with 1ug of CNTN1 protein (10383-H08H, Sino Biological Inc), overnight, at 4°C, prior to application of cultures. Cultures were washed with PBS and fixed with 2% paraformaldehyde for 30 min at room temperature. After washing sequentially with PBS and DMEM (including 20 mM HEPES) cultures were incubated with goat anti-human IgG AF488 (1:750) (A11013, ThermoFisher Scientific) in DMEM/HEPES plus 1% BSA for 1h at room temperature. Cultures were washed sequentially with DMEM/HEPES and PBS and then permeabilised with ice cold methanol (30-45 minutes on ice). Cultures were blocked with 5% NGS in PBS before incubation with primary antibodies chicken anti-Neurofilament 200 (1:10,000) (4680; Abcam) and rat anti-myelin basic protein (1:500) (Ab7349, Abcam) over night at 4°C. Secondary antibody incubation was with biotinylated goat anti-chicken IgY (1:500) (BA9010, Vector Laboratories), goat anti-rat IgG Alexa Fluor 546 (1:1000) (A11081, Life Tech) for 1 hour at room temperature followed by streptavidin pacific blue (1:500) (S11222, Life Technologies) 45 min at RT. Coverslips were mounted onto glass slides with Vectorshield (H1000, Vector) and imaged with confocal microscopy (Zeiss LSM 700).

### Mouse sciatic nerve teased fibres

Sciatic nerves were harvested from a six-week old male C57BL/6 mouse immediately following Schedule 1 killing by rising concentration of CO_2_ and were post-fixed overnight in 4% PFA in 0.1M PBS. For teased fibres the sciatic nerve sheath was removed and smaller nerve bundles transferred to a drop of distilled water on a Superfrost slide. Individual fibres were teased from the bundle and air-dried. Nerve fibres were permeabilised in 100% acetone, 10 min at -20°C, washed 3x in PBS and blocked in 5% fish gelatin in PBS + 0.1% triton-X for 1 hr at room temperature. Patient sera were diluted 1:200 in 0.5% fish gelatin and 0.01% triton-X., including guinea pig anti-Caspr (1:1000)[3] (a gift from Dr Manzoor Bhat, UT Health Science Center San Antonio) and incubated overnight at 4°C in a humidified chamber. The next day slides were washed with PBS and counterstained with goat anti-human IgG Alexa 488 (1:750) (A11013) and goat anti-guinea pig IgG Alexa 568 (1:1000) (A11075, ThermoFisher) in 0.5% fish gelatin and 0.01% triton-X for 1h at room temperature. After washing in PBS, slides were mounted with Vectorshield (H1000, Vector) and imaged with confocal microscopy (Zeiss LSM 700).

## **CNTN1 identification**

### Human samples

Fresh frozen human kidney cortex normal tissue (Request no. 19/A157) and paraffin-embedded kidney biopsy tissues (Request no. 19/A071) were obtained from the Oxford Centre for Histopathology Research (OCHRe) under REC approval number 14/SC/0280. For tissue sections, kidney biopsy samples obtained under local anaesthesia were collected in either phosphate buffer or Michel’s medium, before being placed into formalin (4%) and then processed and embedded according to standards clinical lab protocols. Thereafter, samples were stored at room temperature until needed for cutting. Fresh human kidney tissue was resected from two patients undergoing nephrectomy for renal cell carcinoma. Approximately 0.5 cm^3^ of kidney tissue or cortex were flash frozen in liquid nitrogen and transferred to cryotubes for storage in liquid nitrogen at -80 °C. Sample 1: Whole kidney (Male, aged 63). Sample 2: Whole kidney and cortex (Male aged 65). Additional kidney biopsies were provided by the MRC/Kidney Research UK National DNA Bank for Glomerulonephritis.

Fresh frozen human frontal cortex tissues of normal aged brain from a healthy donor were obtained from the Oxford Brain Bank (Request no. OBB553) under REC approval number 15/SC/0639 (59 year old male; post-mortem delay 1 day; frontal cortex from right hemisphere snap frozen in liquid nitrogen; storage in -80 freezer since May 2013; pathological diagnosis – normal aged brain). Additional formalin-fixed and paraffin embedded healthy human cerebral cortex (HP-210) and kidney tissue sections (HP-901) were purchased from Zyagen (San Diego, CA, USA).

Peripheral blood mononuclear cells (PBMC) were isolated from the whole blood of a healthy donor (40 year old male; venepuncture collected in sodium heparin tubes; Lymphocyte density gradient centrifugation; cryopreservation in 10% DMSO, 40% foetal bovine serum, 50% RPMI; storage in gaseous nitrogen since February 2019).

### Immunohistochemistry

Human kidney diaminobenzidine (DAB) immunohistochemistry for CNTN1. Sections of kidney biopsy tissue from patients 1, 2, 13 and 14 (Supplementary Table 1), as well as PLA_2_R+ patients and healthy human donors, were immunostained in parallel for CNTN1 by DAB immunohistochemistry. Formalin-fixed, paraffin embedded human tissue sections (3-5 µm) sections were rehydrated through two 5 min changes each of xylene, 3 min of 100% ethanol, 1 min of 95% ethanol, and 5 min of 80% ethanol to distilled water. Tissue slides were incubated in near-boiling citrate-EDTA buffer (citric acid 10 mM, EDTA 2·5 mM, tween-20 0·05%, pH 6·2 with NaOH) for 20 min, and after cooling washed with PBS. Blocking and permeabilisation was performed by incubating sections with 10% normal donkey serum (NDS) and 0·3% triton-X in PBS for 1h at room temperature. Primary antibody incubation was with goat anti-CNTN1 (1:800; 2·5 µg/ml) (AF904, R&D systems; lot EPC021808A) overnight at 4°C in 1% NDS and 0·03% triton-X in PBS. The following day after washing in PBS, incubated with horse anti-goat biotin (1:200) (BA9500, Vector labs) in 1% NDS and 0·03% triton-X in PBS for 90 minutes at room temperature. After washing in PBS, endogenous peroxidase activity was quenched by 3% H_2_O_2_ in PBS for 30 min. Sections were washed in azide-free PBS and incubated with ABC reagent (Vectorstain Elite ABC HRP kit, Vector, PK-6100) 30 min at room temperature, followed by wash with distilled water. Sections were developed by incubating with DAB reagent (SK-4100, DAB peroxidase substrate Kit, Vector) 1-5 min and stopped stain by rinsing in water. Sections were counterstained with haematoxylin (Mayer’s) solution (51275, Sigma), dehydrated back through alcohol series with two changes each: 95% ethanol (30 s), 100% ethanol (30 s), xylene (30 s) and mounted with DPX and coverslip. Sections were imaged on an upright bright-field microscope fitted with digital camera. CNTN1 antibody concentration was established empirically prior to experiments by titration on positive control tissue (healthy human brain cortex) (HP-210, Zyagen) and compared with staining where the primary antibody was omitted.

### Western Blot

#### Cell line Lysate preparation

CNTN1-transfected HEK cells and iPSC-derived neurons were cultured as described, washed once in PBS, lifted into suspension with TrypLE Express (12604-021, Gibco) and centrifuged at 500g for 5 minutes. The pellets were resuspended in RIPA buffer (R0278, Sigma) and Halt protease inhibitor cocktail (1:100) (1862209, ThermoFisher Scientific), rested on ice for 15 minutes before re-pipetting to homogenise. After a further 30 minutes with gentle inversion at 4°C and re-pipetting to homogenise they are centrifuged at 4°C for 5 minutes at 10g. Protein quantification of the supernatant (for cell line and all tissue lysates) was determined using the Pierce™ BCA protein assay kit (23225, ThermoFisher scientific) prior to storage at -80°C until required for use in western blot.

#### Rat tissue lysate preparation (brain, kidney, sciatic nerve)

Rat tissue was harvested from a six-week old male Wistar rat immediately following Schedule 1 killing by rising concentration of CO_2_. Dissected tissues were immediately frozen on dry ice stored at -80°C. For lysis, tissues were weighed, cut into ~1mm sections, then incubated with RIPA buffer and protease inhibitor cocktail (1:100) (300μl lysis buffer per 0·1g tissue) at 4°C for 20 minutes with gentle inversion. Tissues were homogenised on ice with a handheld homogeniser and lysates were centrifuged at 10,000g for 5 minutes at 4°C prior to transfer of supernatant.

#### Human tissue lysate preparation (podocyte, whole kidney)

Cortical tissue from fresh frozen healthy human kidney was dissected into two 200 mg pieces, each transferred to 2ml microcentrifuge tubes (QIAGEN) on ice and incubated with 1ml of RIPA buffer, Pepstatin A and 1:100 Halt™ PIC. A 5mm steel bead (QIAGEN) was added to each tube and samples were homogenised on ice at 30Hz using a tissue homogeniser for a total of 10 minutes, with 2 minute cycles at 1 minute intervals. The homogenates were placed on a rotator at 4°C for 2 h before being centrifuged at 5100g at 4°C for 30 minutes. The supernatant was transferred to a clean tube and centrifuged again at 15800g 4°C for 15 minutes. This final supernatant was stored at -20°C until further use in western blotting.

Human podocytes from an immortalised cell line (kindly provided by Professor Moin Saleem, University of Bristol) were grown in Roswell Park Memorial Institute (RPMI) 1640 medium (Cat no. 31870-025, Gibco), 1% Insulin-Transferrin-Selenium (ITS) (Cat no. 41400-045, Gibco), 10% FBS, 1% Penicillin Streptomycin and 5mM L-glutamine at 33°C , the medium only supplemented with the latter when the cells were passaged (twice weekly) or medium was changed (three times weekly). They were subsequently incubated at 37°C for two weeks to allow for differentiation into podocytes. These were washed in PBS, lifted with Trypsin-EDTA (0·05%) and centrifuged at 200 x g for 5 minutes. The pellet was resuspended thoroughly in RIPA buffer and Halt PIC and kept on ice for 15 minutes before further homogenisation and centrifugation at 4°C at maximum speed for 15 minutes. The supernatant was collected and transferred to a clean sample tube for storage at -80°C.

#### Gel electrophoresis and protein detection

Cell lysates, tissue homogenates, and human recombinant CNTN1 protein (10383-H08H, Sino Biological) were reduced and denatured by mixing with sample reducing agent, (B0009, Thermofisher) and LDS sample buffer (B0008, Thermofisher) before heating at 70°C for 15 mins. They were loaded (5-60µg rat tissue, 40µg podocyte fraction or 30µg whole kidney including glomeruli for human kidney lysates) onto an 8% polyacrylamide gel (Bolt Bis-tris plus; ThermoFisher Scientific) for electrophoresis and run at 150V 400mA on a mini gel tank (A25977, Thermofisher) . Separated proteins were transferred onto a nitrocellulose membrane (LC2000, ThermoFisher) and blocked with 5% non-fat milk in Tris-buffered saline plus 0·1% Tween 20 (TBS-T) before incubation with either goat anti-CNTN1 (1:12,000) (AF904, R&D Systems) (Note: This antibody is cross-reactive to human, mouse and rat CNTN1) or CNTN1 antibody-positive patient sera from patients 1-4, 13 and 16 at 1:500, and P14 at 1:100, in milk, overnight at 4°C. P16 was additionally pre-incubated with recombinant CNTN1 protein prior to gel electrophoresis to demonstrate abrogation of CNTN1 reactivity and antibody specificity. Secondary antibody incubation was with either peroxidase conjugated anti-goat IgG (705-036; Jackson ImmunoResearch; 1:2000) or anti-human IgG4 (MH1742, Thermofisher), respectively. Protein detection was performed using ECL Prime detection reagent (RPN2232, GE Healthcare).

*Serum immune complex precipitation*

Polyethylene glycol (PEG) 6000 (Cat no. 81260, Sigma) was dissolved in borate buffer to make a 12.5% stock solution, and after briefly mixing whole sera with 50ul borate buffer and 50ul 0.2M EDTA, 100ul was added to make a final concentration of 2.5% and left overnight at 4°C to precipitate immune complexes as previously described.[4] After centrifugation at 2000g for 30 minutes at 4°C, pellets were resuspended in 1ml of 2.5% PEG before centrifugation again. PEG precipitates were resuspended in 30ul of 37°C prewarmed PBS and loaded onto SDS-PAGE gel for electrophoresis and western blotting (as above). Protein was transferred to a nitrocellulose membrane, blocked with 5% non-fat milk in Tris-buffered saline plus 0·1% Tween 20 (TBS-T) and blotted with goat anti-CNTN1 (1:1,000) (AF904, R&D Systems) followed by peroxidase conjugated donkey anti-goat IgG (1:20,000) (705-036; Jackson ImmunoResearch). Protein detection was performed using ECL Prime detection reagent (RPN2232, GE Healthcare).

### Serum CNTN1 protein measurement

Serum CNTN-1 protein levels were measured on the Luminex® platform according to the manufacturer’s instructions (Human Magnetic Luminex Assay, R&D systems) as previously described.[5] Samples were coded randomly and analysed in duplicate. Measurements with a coefficient of variation >15% and outliers were repeated.

### End-point polymerase chain reaction (PCR)

Frozen human brain and kidney tissues (40-80 mg wet tissue) were incubated overnight in RNA Later Ice (Cat no. 4427575, Thermo Fisher) at -20°C prior to homogenisation by rotor-stator and trituration through a sterile needle and syringe on ice. Cryopreserved human PBMC were thawed, washed in PBS and pelleted prior to lysis. RNA was extracted with TriPure reagent (500 µl per sample) and isolated using a High Pure RNA Isolation kit (Cat no. 11 828 665 001, Roche) according to the manufacturer’s instructions. All cDNA prepared by reverse transcription from 250 ng extracted RNA using Evoscript Universal cDNA Master (Cat no. 07 912 374 001, Roche). Omission of reverse transcriptase was used as a negative control (-RT) for genomic DNA contamination. For PCR, cDNA were diluted 1:10 and used at 1 µl in a 20 µl reaction volume with GoTaq G2 Flexi DNA Polymerase (M7801, Promega) using custom designed primers (10 µM). Two sets of primers per target transcript were validated using a cDNA library constructed from RNA isolated from the lysate of HEK cells transiently transfected with the corresponding transgene (See Supplementary Table 2 for sequences of primers used in the paper). PCR products were separated by electrophoresis in 1·5% agarose gel stained with Safe Pinky (20 min at 140V) and visualised on a UV illuminator. Correct amplification of transcript cDNA was confirmed by a single band at the predicted size relative to a 100 bp DNA ladder marker. Primer sequences for PCR reactions were as follows: *CNTN1* (NM_001256063.1) *Forward* 5’- TGCAAATGTTGAGGCTTCCG -3’, *Reverse* 5’- TCCGGAACAGGATTTCCAAGT -3’(product size 222 bp); *NFASC* (NM_001005388.2, NM_001160331.1) *Forward* 5’- AACGCCTTTGTCAGTGTGCT -3’, *Reverse* 5’- TAGTTGCCACCATCCAGGTT -3’ (product size 176 bp); *NPHS2* (NM_014625.3) *Forward* 5’- TGTGCAAACCACTATGAAGCG-3’, *Reverse* 5’- TTCTGCAGCAATCATCCGCA -3’(product size 238 bp); *YWHAZ* (NM_003406.3) *Forward* 5’ CCTGCATGAAGTCTGTAACTGAG -3’, *Reverse* 5’- GACCTACGGGCTCCTACAACA -3’ (product size 100 bp).

### Laser microdissection and mass-spectrometry proteomic analysis

Three formalin-fixed paraffin embedded renal tissues, one positive for CNTN1 by immunostaining and two negative controls, together with two positive control brain tissues were laser micro-dissected and captured using the Leica LDM7 system. The samples, together with recombinant human contactin-1 (10383-H08H, Sino Biological) were analysed by a proteomics approach.[6] Samples were solubilised into 10mM Tris in MilliQ water containing 1mM EDTA and 0.002% Zwittergent, heated at 99 °C for 90 min and then sonicated for 60 min. Proteins were digested with Trypsin Gold (30 ng) overnight at 37 °C in a thermoshaker, reduced with dithiothreitol (50 µg) at 99 °C for 5 min; and samples were dried using a SpeedVac at room temperature. Peptide mixtures were re-suspended in 0·1% trifluoroacetic acid in water (20 µl) and analysed by LC-MS/MS, using a Thermo Scientific Q-Exactive Plus mass spectrometer coupled to a Dionex Ultimate 3000 nanoLC system equipped with a Thermo Easy-spray Acclaim Pepmap column (75μm x 15cm, 3μm/100Å packing). Peptides elution was performed using a flow rate of 300 nl/min with a 30 min linear gradient of acetonitrile:water:formic acid (5:95:0.1 – 56:44:0.1 v/v/v). A full MS scan (mass range: m/z 350-1400) was acquired with a maximum injection time of 100 ms, 70,000 of resolution and the 10 most intense precursor ions with a minimum intensity of 2·0e4 were selected for higher-energy C-trap dissociation (HCD). The normalised collision energy was set at 28, the isolation width and the dynamic exclusion were 2 m/z and 20 s respectively; mono-charged ions were excluded. MS raw data were analysed by Mascot software (Matrix Science, London, UK) using the Swiss-Prot human database. Mascot search parameters were set as follow: trypsin as proteolytic enzyme, two missed cleavages, precursor mass tolerance of 10 ppm, 0·6 Da for HCD fragments, methionine oxidation as variable modification; charge states +2, +3 and +4 and a significance level at p<0·05.

# **SUPPLEMENTARY FIGURES**


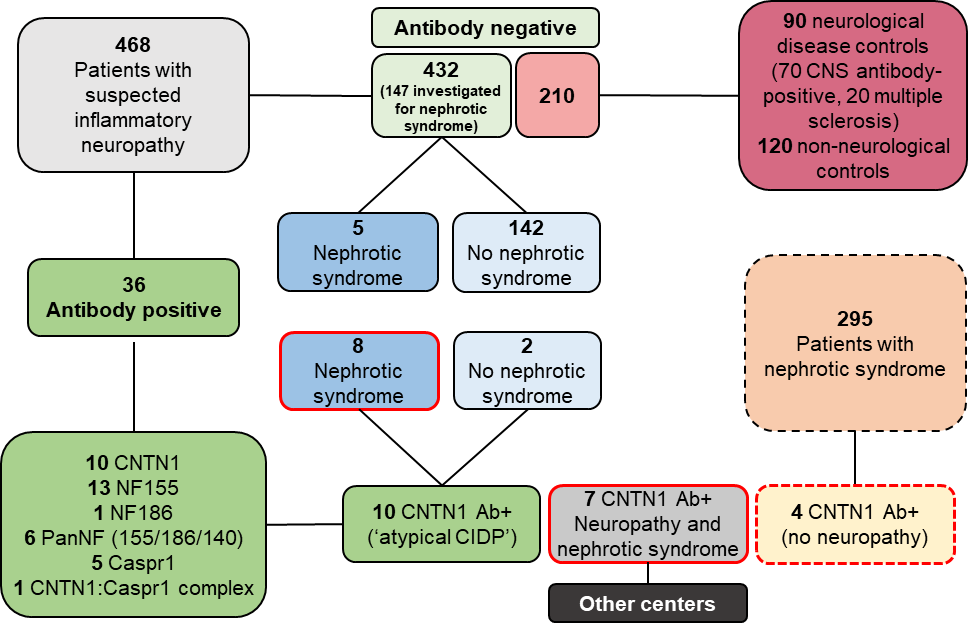


**Figure S1. Flowchart summarising detection of patients with CIDP, nephrotic syndrome and CNTN1 antibodies through prospective nodal/paranodal autoantibody screening.**

Detection of 8 patients with definite CIDP, nephrotic syndrome and CNTN1 antibodies through prospective testing of sera from patients with suspected inflammatory neuropathies. Subsequently, seven patients were identified from other testing centres, and sera from a further 295 patients with known nephrotic syndrome caused by idiopathic membranous glomerulonephritis were tested only for CNTN1 antibodies using cell-based assay, and 4 were weakly positive (IgG fluorescence labelling to CNTN1-treansfected cells clearly visible, but weakly above background). Patients with central nervous system (CNS) autoimmunity, including 70 antibody-mediated disorders (n=21 anti-aquaporin 4, n=9 anti-NMDA receptor, n=20 anti-glycine receptor, n=20 anti-myelin oligodendrocyte glycoprotein) were used as neurological controls; all were negative for NF186/140, NF155, CNTN1 and Caspr1 antibodies, as were all of the 120 non-neurological controls, which included healthy individuals and those with uncomplicated viral infections, and all 46 patients with secondary membranous lupus nephritis. NMDA, N-Methyl-D-aspartate. Sera from an additional seven patients with neuropathy and nephropathy were provided by other centres. Patients with anti-CNTN1 antibodies, neuropathy *and* nephropathy are shown in boxes outlined in red; dashed line indicates lack of diagnosed neuropathy.

A

B

**
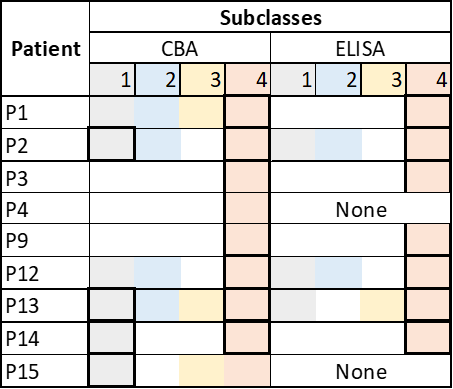
**

C

**Figure S2. Titre and subclass specificity comparison between CBA and ELISA**

(A) The average end-point titre for CNTN1 antibody detection was slightly higher using ELISA compared with CBA, though antibodies were detected in all the 9 patient sera samples available locally for testing using both assays (titre for P09 was only tested on ELISA so no data point is shown for CBA). The mean titre is indicated by the lines on left-hand graph, and lines linking individual patients’ results across 2 assays can be seen on the right-hand graph. There were 2 patients with titres of 3200 on CBA (both 1600 on ELISA) and 2 with titres of 800 on CBA, thus these data points are merged. (B) For the same 9 patients, antibody subclass testing revealed specificity mainly to IgG4 in both assays, but often with the presence of other subclasses. The predominant subclass, assessed by visual scoring of the fluorescence intensity of IgG binding to transfected cells in CBA, is indicated by a boldly outlined box. IgG1 was deemed to be the predominant subclass in P15, and equally dominant in P2, P13 and P14. No subclass was detected on ELISA for P4 and P15. (C) Optical density (OD) is shown as a quantitative measure for each IgG subclass and panIgG in the ELISA for the same patients.

**Figure S3. Immunoreactivity of IgG from CNTN1-positive patients on CBA show they are specific for Contactin-1 (CNTN1).**

IgG from CNTN1-positive sera react with only CNTN1-transfected HEK cells (top panel; commercial CNTN1 stain in red, patient IgG green, cell nuclei in blue). Further, reactivity is abrogated or significantly diminished when pre-adsorbed with CNTN1 protein (middle panel). No reactivity is seen following incubation with untransfected cells (bottom panel). These images were obtained using sera from P2, and reflect similar results obtained for all sera available for further testing from the cohort (P1, P3, P4, P12, P13, P14 and P15).

**
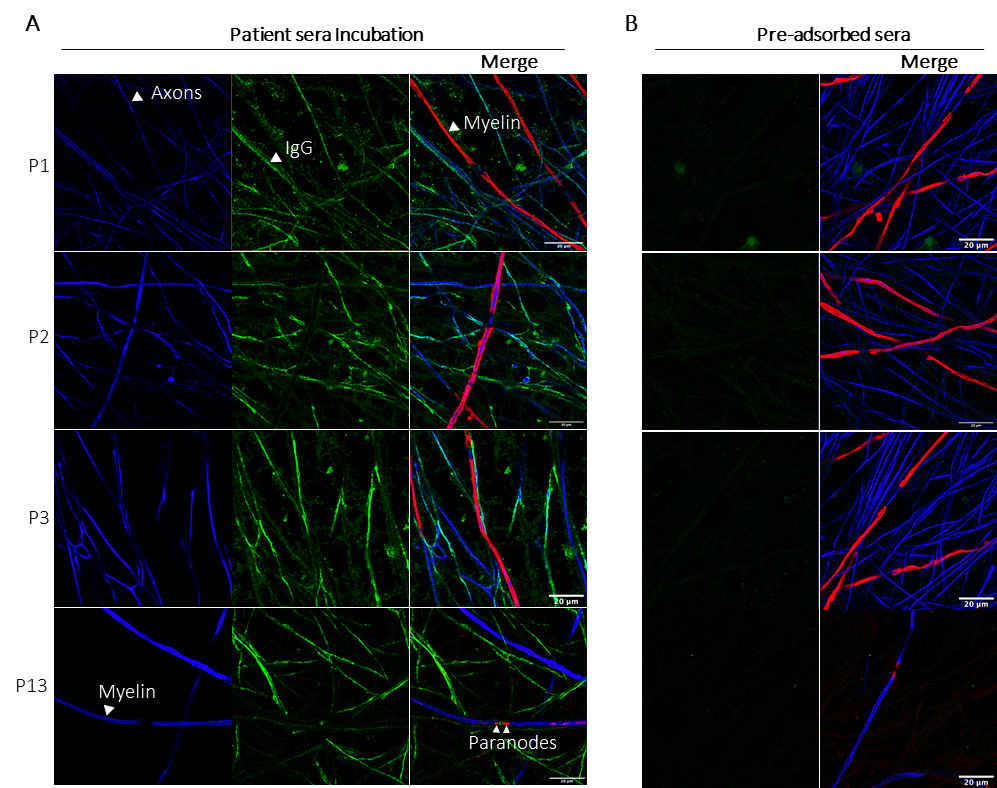
**

**Figure S4. Axonal pattern of IgG binding from CNTN1-positive patients in myelinated neuronal co-cultures is specific to the CNTN1 antigen**

Patient sera tested on myelinated co-cultures were available for nine patients (four shown above). Sera for P4 was available but tested negative for IgG reactivity against neurons, and is not shown. Sera for P09 was only available at the time of initial testing, and not pre-adsorption experiments. A) Human IgG binding (green immunofluorescence, middle column) to human iPSC-derived axons (blue, immunolabelled with Neurofilament antibody NF200, first column) is demonstrated for P1-P3, and P13. These can be seen to co-localise (merged image, third column), but not with myelin (red, immunolabelled with myelin basic protein – MBP). For P13 there was some additional IgG labelling at the node. This can be better appreciated, as shown, with paranodes labelled with anti-caspr antibodies (red), and myelin in blue. Images showing independently marked are not shown. B) Sera were pre-adsorbed with CNTN1 protein and re-applied to co-cultures. Patient IgG binding was abrogated in all cases tested.


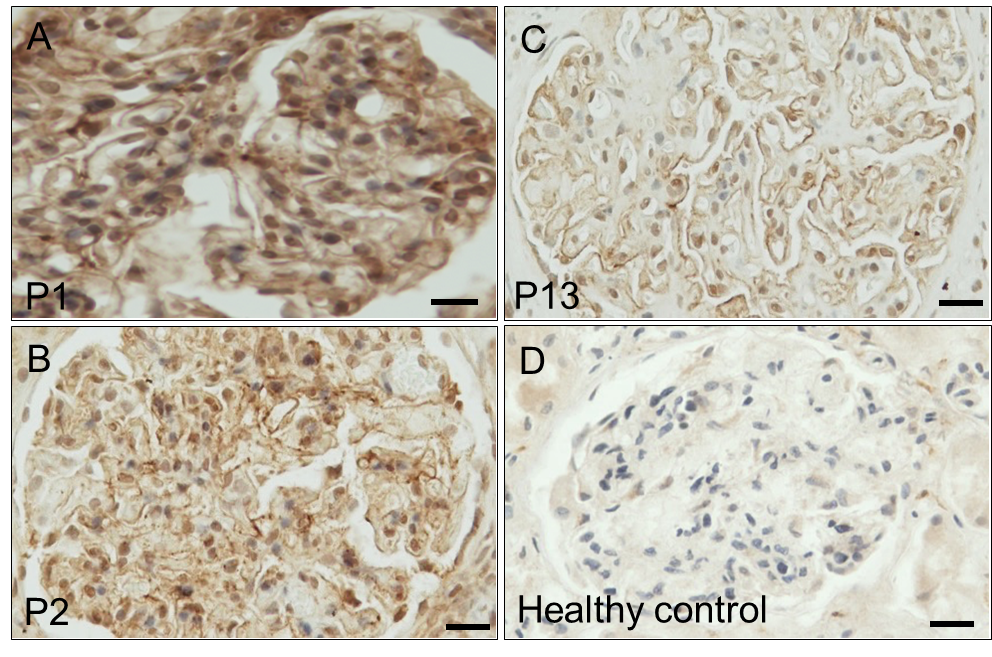


**Figure S5. Contactin-1 immunoreactivity in kidney immune deposits for patents 1, 2 and 13**

Diaminobenzidine immunohistochemistry for CNTN1 is observed along the basement membrane of glomeruli from patients 1, 2 and 13 in (A), (B) and (C), respectively. D) No positive CNTN1 staining can be seen in a healthy control kidney specimen. Scale bars 20µm.

**
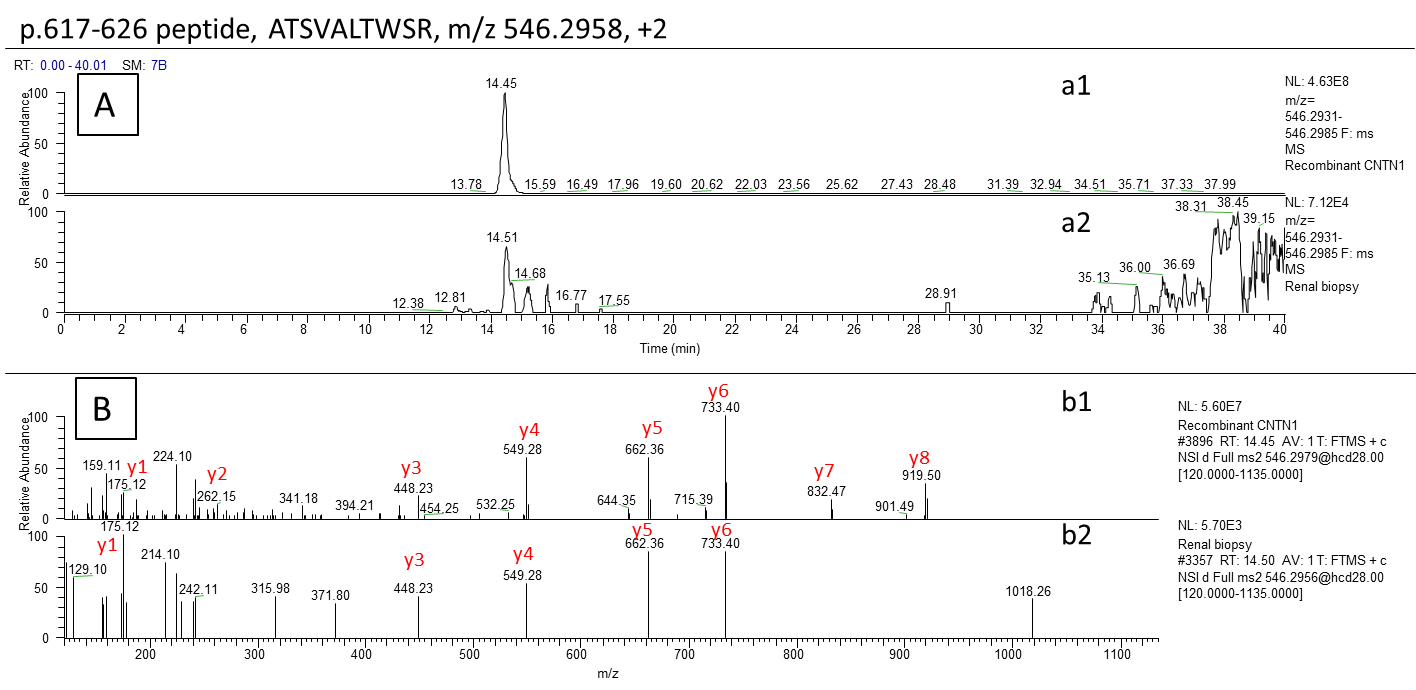
**

**Figure S6. LC-MS/MS of CNTN1 peptides. A**) Extracted-ion chromatograms for p.617-626 peptide (MH_2_^2+^ m/z 546.2958) from recombinant CNTN1 (*above*, a1) and laser-dissected CNTN1-immunopositive kidney biopsy (*below*, a2). The p.617-626 peptide elutes ~ 14.5 min in both samples. **B**) HCD mass spectra for p.617-626 peptide from recombinant CNTN1 (*above, b1*) and CNTN1-immunopositive kidney biopsy (*below, b2*). HCD spectra with a prevalence of y series fragment ions confirming the identity of the peptide.

**
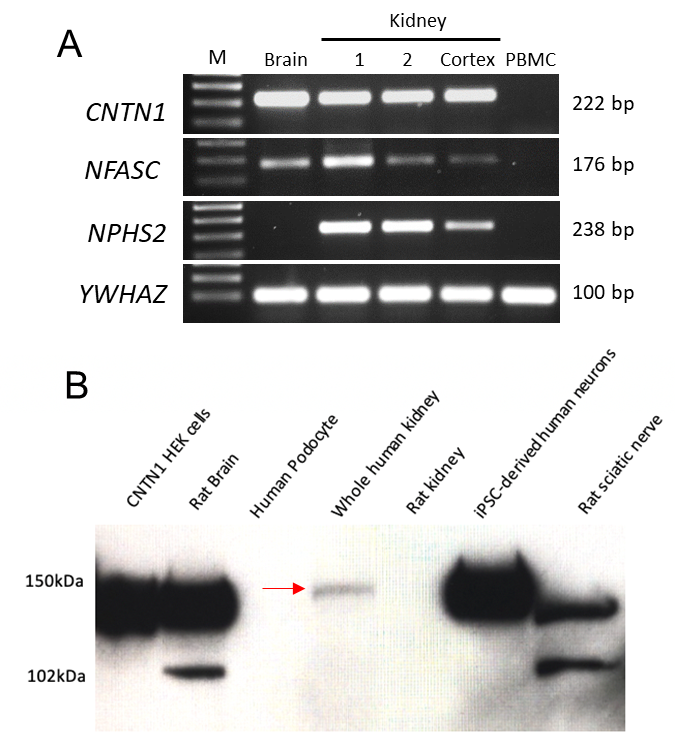
**

**
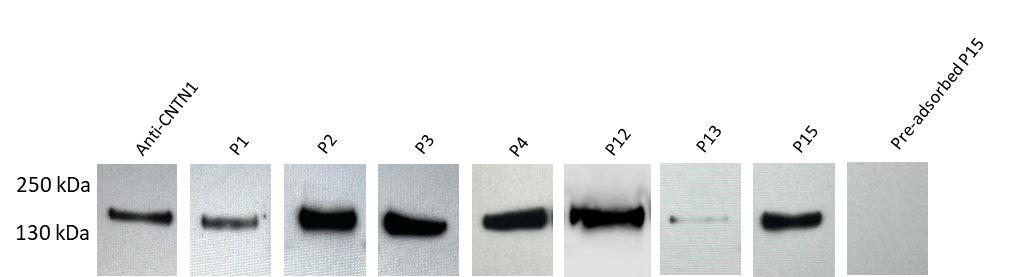
**

C

**Figure S7. CNTN1 is expressed in healthy human kidney. A**) Gel electrophoresis of contactin 1 (*CNTN1*), pan-neurofascin (pan) (*NFASC*), podocin (*NPHS2*) and house-keeping gene 14-3-3 protein zeta/delta (*YWHAZ*) PCR products in human brain, kidney (whole tissue lysate from two patients, as well as isolated cortex) and peripheral blood mononuclear cells (PBMC) from a healthy donor. **B**) Western blot of human and rat tissue lysates with anti-CNTN1 antibody showing weak detection of CNTN1 protein in healthy human kidney. Faint band around 150 kDa in whole human kidney lysate (arrow, lane 4) corresponds to prominent bands in positive control samples for CNTN1 detection: lysate of HEK cells transfected with untagged CNTN1 (lane 1), rat brain (lane 2), human iPSC-derived sensory neurons (lane 6) and rat sciatic nerve (lane 7). An additional band is detected around 100 kDa in rat brain and sciatic nerve tissue, which may represent an alternative isoform of CNTN1. No bands are seen in lysate of a differentiated human podocyte cell line or rat kidney. Protein loading: CNTN1 transfected HEK cells (30µg); rat brain (5µg); podocyte lysate (40µg); healthy human kidney (30µg); iPSC-derived sensory neurons (40µg); rat sciatic nerve (60µg). **C**) Western blot shows bands at approximately 150kDa when patient sera (P1, 2, 3, 4, 12, 13 and 15) is used as a primary antibody against recombinant CNTN1 protein loaded onto the gel. As all sera contain predominantly IgG4 subclass antibodies, anti-IgG4 HRP was used as a secondary antibody against sera. These bands align with the band produced when a commercial anti-CNTN1 antibody (lane 1) is used as a primary antibody, and can be abrogated by pre-adsorbing the sera with CNTN1 protein (lane 9), confirming the antibodies are specific for CNTN1.


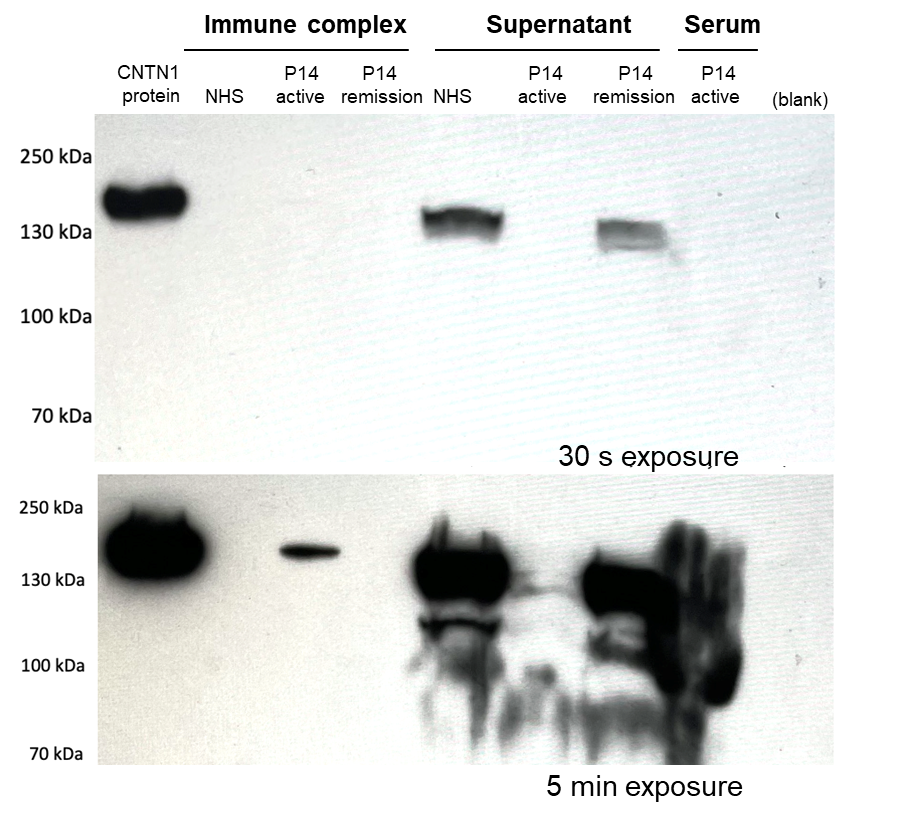


**Figure S8. CNTN1-containing immune complexes are present in serum at disease nadir.** Repeat of full western blots at two different exposure times**:** 30s (above) and 5 min (below). Gel was loaded with 5µl of precipitate (immune complexes) or 1µl of the remaining supernatant purified from 300µl serum; 5µl of whole sera was also loaded. Recombinant CNTN1 protein loading, 0.01 µg.

**TABLE**

| **PATIENTS** | **P1** | **P2** | **P3** | **P4** | **P5** | **P6** | **P7** |
| --- | --- | --- | --- | --- | --- | --- | --- |
| **Demographic** | 56 M | 49 M | 39 M | 50 M | 79 M | 74 M | 62 M |
| **PMH/FH** | -T2DM  -Bilateral carpel tunnel  -Pulmonary emboli  -Two 1^st^ degree relatives:  diabetes  -Two sisters: HTLV-1  -1^st^ degree relative:  Lupus | -HTN  -Gout  -1^st^ degree  relative:  GBS | -T2DM  Optic neuritis (OPA1 mutation)  -15 years relapsing-remitting painful paraesthesia (tongue, hands and feet)  and co-existent MGN | None | None | None | -BPH  -Hyperlipidaemia  -Pulmonary emboli |
| **Neuropathy**  **onset** | Subacute | Subacute | Subacute  on chronic | Subacute | Subacute | Subacute | >2 months to nadir, rapid progression |
| **Limb symptoms**  **(15/16 ‘definite CIDP’)*** | Typical phenotype†  (proximal ULs, distal LLs predominant) | Typical  phenotype | Typical  phenotype  (deterioration in sensory symptoms with mild weakness) | Typical  phenotype  (paraesthesia ULs followed 3 months later by weakness all limbs) | Typical  phenotype | Typical  phenotype | Typical  phenotype |
| **Additional clinical features** | Postural tremor  Abdominal bloating & weakness** | Back pain  Sensory ataxia  Abdominal bloating & weakness** | Neuropathic pain  Postural tremor  (ULs, voice, head) | Dysphonia  Unilateral ptosis  Gait ataxia | Facial palsy | Neuropathic  Pain  Sensory ataxia | Neuropathic pain  Autonomic disturbance  (Hyperhidrosis and constipation) |
| **Nadir**  **Disability** | Mild  (mRS 2) | Moderate  (mRS 3) | Moderate  (mRS 3) | Mod-severe  (mRS 4) | Death  (mRS 6) | Death  (mRS 6) | Mod-severe  (mRS 4) |
| **Notable blood results** | ↑ ESR | ↑ ESR 109mm/hr  IgA Kappa paraprotein | NA | NA | NA | NA | NA |

| **PATIENTS** | **P1** | **P2** | **P3** | **P4** | **P5** | **P6** | **P7** |
| --- | --- | --- | --- | --- | --- | --- | --- |
| **CSF WCC** | <5 | <5 | <5 | ↑ 28  (100% monocytes) | <5 | <5 | ↑ 14 |
| **CSF Protein g/l** | 0.32 | ↑ 2.1 | 0.24 | ↑ 2.8 | ↑ 2.4 | ↑ 1.5 | ↑ 2.08 |
| **Imaging** | MRI neuroaxis/ CT CAP/  PET  normal | MRI neuroaxis/  CT CAP normal | ND | MRI neuroaxis/  PET-CT  normal | ND | ND | MRI neuroaxis normal |
| **Clinical**  **Evidence of nephrotic syndrome/renal dysfunction §** | Within 5 months  Oedema  ↓ albumin (11g/L)  ↑ PCR  (1142mg/  mmol) | Within weeks  (No oedema)  ↓ albumin (24g/L)  ↑ PCR  (633mg/  mmol) | Within 2 months  Oedema  ↓ albumin  (26g/L)  Proteinuria  (3.5g/24hr)  ↑ PCR  (1032mg/  mmol) | Within a month  Oedema  ↓albumin  (12g/L)  ↑ PCR  (4638mg/  mmol) | Oedema  Creatinine 100µmol/L  Proteinuria  (3g/24hr)  PCR (180mg/mmol) | Albumin (36g/L)  Creatinine 87µmol/L  Proteinuria (3.35g/24hr)  ↑ PCR  (718mg/  mmol) | Oedema  Creatinine stated to be within ‘normal range’  ↓ albumin  (20g/l)  Proteinuria (8.3g/24hr)  ↑ PCR |
| **Renal**  **Biopsy** | MGN  Positive: IgG, C3  Negative:IgA, C1q | MGN  Positive: IgG, C3, IgA  Negative: IgM, C1q | MGN  Positive: Immune complex deposition, IgG, C3 | MGN  Positive: IgG, C3/4  Negative: IgA, C1q, IgM | MGN | MGN | ND |
| **PLA_2_R status** \|\| | Negative IHC | Negative IHC | ND | Antibody negative | Antibody negative | Antibody negative | ND |
| **Treatment**  **(response if clear** | Concurrent steroids and  CYC  (good) | Steroids  (good)  IVIg  (partial) | Steroids  (partial)  IVIg  (good)  Tacrolimus  (good)  Thalatomy  (for tremor) | Steroids  IVIg  PE  CYC  RTX  (unresponsive to all above)  ASCT  (Good) | Steroids  IVIg  PE | Steroids  IVIg  PE  RTX | Steroids  IVIg  AZA  PE  (unresponsive to all above)  RTX  (good) |

| **PATIENTS** | **P1** | **P2** | **P3** | **P4** | **P5** | **P6** | **P7** |
| --- | --- | --- | --- | --- | --- | --- | --- |
| **OUTCOME**  **(neuropathy)** | Complete remission  (mRS 0)  (Steroid  dependent) | Good  (mRS 1) | Good  (mRS 1) | Good  (mRS 2) | Death (Multi-organ failure) | Death (Multi-organ failure | Complete remission  (mRS 0) |
| **OUTCOME**  **(nephropathy)** | Stabilised | Complete remission | Complete remission |  |  |  |  |

**Table S1. Key characteristics of 15 patients with immune-mediated neuropathy and nephrotic syndrome with positive anti-CNTN1 antibodies (patients 1-7)**

* According to EFNS/PNS criteria

** Associated with severe denervation of rectus abdominis and iliopsoas on EMG when tested for in one patient

† Symmetrical non-length dependent weakness and sensory disturbance in all limbs

§ ‘nephrotic’ range (PCR > 300mg/mmol or proteinuria >3g/24hr), suggestive of glomerular pathology (PCR >100mg/mmol), normal serum albumin (35-55g/l). Proteinuria is in grams over a 24 hour period. Timing relative to neuropathy stated where available.

|| IgG subclass was only available for one patient (P13)

# PLA_2_R was specifically tested for only where status is confirmed

**Abbreviations:**

ACR – Albumin: Creatinine ratio, AS – Aortic Stenosis, ASCT – Autologous Stem Cell Transplant, AZA – Azathioprine, BPH – Benign prostatic hyperplasia, CKD – Chronic kidney disease, CT CAP – Computed tomography chest abdomen pelvis, CYC – Cyclophosphamide, ESR – Erythrocyte sedimentation rate, FH – Family history, HTN – Hypertension, HTLV-1 – Human T-lymphocytic virus 1, IA – Immunoadsorption, IDDM – Insulin dependent diabetes mellitus, IHC – Immunohistochemistry, ITP -Idiopathic Thrombocytopenic Purpura, IVIg – Intravenous Immunoglobulin, LL – Lower limb, MGN – Membranous Glomerulonephritis, MMF – Mycophenolate Mofetil, MRI – Magnetic resonance Imaging, mRS – Modified rankin score, ND – Not done, OPA1 – Optic atrophy type 1, PCR – Protein:Creatinine Ratio, PE - Plasma Exchange, PET – Positron emission tomography, RTX – Rituximab, T2DM – Type two diabetes mellitus, UL – Upper limb, WCC – White Cell Count

| **PATIENTS** | **P8** | **P09** | **P10** | **P11** | **P12** | **P13** | **P14** | **P15** |
| --- | --- | --- | --- | --- | --- | --- | --- | --- |
| **Demographic** | 58 M | 62 M | 66 M | 39 F | 52F | 60 F | 72 M | 59 M |
| **PMH/FH** | Graves’ disease | -IDDM  -HTN  -BPH | None | None | Two 1^st^  degree  Relatives  - diabetes | -T2DM  -HTN  -Hashimoto’s thyroiditis  -ITP  -Severe AS  -2 year history  progressive tremor and  gait disturbance  (diagnosed CIDP 2015) | -T2DM  -HTN  -Chronic pancreatitis  -Hyperlipidaemia  -CKD (baseline ACR 35) – obstructive uropathy and recent bladder neck surgery | None |
| **Neuropathy onset** | Subacute | Subacute | Subacute | Subacute | Subacute | Subacute on chronic | Subacute | Chronic  progressive |
| **Limb symptoms**  **(15/16 ‘definite CIDP’)*** | Typical  phenotype | Typical  phenotype | Typical  phenotype | Typical  phenotype | Typical  phenotype | Typical  phenotype | Typical  phenotype | Typical  phenotype  (proximal-dominant weakness) |
| **Additional clinical features** | Sensory  ataxia | -Back pain  -Facial paralysis  -Autonomic disturbance  (Urinary urgency,  orthostatic hypotension) | -Facial palsy  -Tremor | -Sensory Ataxia  -Tremor | -Sciatic-like pain  -Sensory  ataxia | -Tremor  -Gait ataxia | Back pain | -Neuropathic pain  -Sensory ataxia  -Autonomic disturbance  -Rash  -Hyper-somnolence  -REM-BD  -Cognitive decline  -Constitutional symptoms (sweats, weight loss) |
| **Nadir**  **Disability** | Severe  (mRS 5) | Severe  (mRS 5)  Tetraparesis,  Required artificial ventilation | Severe  (mRS 5) | Mild  (mRS 2) | Severe  (mRS 5) | Moderately severe (mRS 4) prior to death due to COVID19 | Severe  (mRS 5)  Tetraparesis,  Required artificial ventilation | Moderately severe  (mRS 4) |

| **PATIENTS** | **P8** | **P09** | **P10** | **P11** | **P12** | **P13** | **P14** | **P15** |
| --- | --- | --- | --- | --- | --- | --- | --- | --- |
| **Notable blood results** | NA | ↑ ESR  >120mm/hr | NA | NA | ↑ Eosinophils | NA | NA | ↑ ESR, WCC, eosinophils, serum IgG4 |
| **CSF WCC** | <5 | <5 | <5 | <5 | <5 | ND | ↑ 40  (100% lymphocytes) | ↑ 5-8 |
| **CSF Protein g/l** | ↑ 1.4 | ↑ 1.9 | 0.46 | ↑ 2.25 | ↑ 3.14  (CSF/Serum albumin ratio 69.64) | ND | ↑ >6 | ↑ 0.99 |
| **Imaging** | Lumbar spine MRI: mild enlargement cauda equina nerve roots (no gadolinium) | ND | ND | ND | MRI Brain, Spine and plexi enhancement and thickening of roots  PET-CT – lymph  nodes ‘reactive’ | ND | CT CAP  (enlarged para aortic lymph node) | MRI brain and spine, CT CAP unremarkable |
| **Clinical**  **Evidence of nephrotic syndrome/renal dysfunction §** | Within 2 months  Oedema  Creatinine 55-96mol/L  ↓ albumin  Proteinuria  (10g/24hrs) | Creatinine 60-67mol/L  ↓ albumin (12g/l)  ↑ PCR (716  mg/mmol) | Creatinine 82µmol/L  PCR (220mg/mmol)  Proteinuria (2.2g/24hrs) | Not available | Proteinuria | Oedema  ↓ albumin (27g/l)  ↑ PCR  (1300mg/  mmol) | ↓albumin (18g/l)  Proteinuria  (3.05g/24hrs)  ↑ PCR (1015mg/mmol) | ↓ albumin (24g/l)  Proteinuria  (8.4g/24hrs) |
| **Renal**  **Biopsy** | MGN  Positive: IgG, C3 | MGN and  Diabetic nephropathy  Positive: IgG, IgA | MGN  Positive: IgG,  C3/4 | MGN | MGN  Positive: IgG  Negative: IgM, IgA, C3, C1q | MGN  Positive: IgG4, C1q | ND | ND |
| **PLA_2_R status** \|\| | ND | Antibody negative | Antibody negative | Antibody negative | Negative IHC and  antibodies | Negative IHC and antibodies | ND | ND |

| **PATIENTS** | **P8** | **P09** | **P10** | **P11** | **P12** | **P13** | **P14** | **P15** |
| --- | --- | --- | --- | --- | --- | --- | --- | --- |
| **Treatment**  **(response if clear)** | IVIg  (mild)  Steroids  (none)  CYC and PE  (good) | Steroids  IVIg  PE  (unresponsive to all above)  RTX  Protein A (IA)  CYC | Steroids  IVIg  PE  RTX | Steroids  IVIg  AZA  MMF | Steroids  (more effective than IVIg)  IVIg | Steroids  IVIg  (transient)  RTX | IVIg  (transient)  Steroids  (none)  PE  (none)  RTX  (good) | Steroids  (good) |
| **OUTCOME**  **(neuropathy)** | Complete remission  (mRS 0) | Good  (mRS 3) | Stabilised  (mRS 4) | Complete remission  (mRS 0) | Good  (mRS 1) | Death  (COVID19)  Prior treatment not effective | Good  (mRS 2) | Significant and spontaneous resolution followed by relapse, successfully treated with steroids  (mRS 0, lost to follow up) |
| **OUTCOME**  **(nephropathy)** |  | Good |  | Stabilised | Good |  | Good |  |

**Table S1. Key characteristics of 15 patients with immune-mediated neuropathy and nephrotic syndrome with positive anti-CNTN1 antibodies (patients 8-15)**

# **REFERENCES**

1. Delmont E, Manso C, Querol L, Cortese A, Berardinelli A, Lozza A, et al. Autoantibodies to nodal isoforms of neurofascin in chronic inflammatory demyelinating polyneuropathy. Brain. 2017;140(7):1851–8.

2. Chambers SM, Qi Y, Mica Y, Lee G, Zhang XJ, Niu L, et al. Combined small-molecule inhibition accelerates developmental timing and converts human pluripotent stem cells into nociceptors. Nat Biotechnol [Internet]. 2012 Jul 1 [cited 2019 Mar 29];30(7):715–20. Available from: http://www.ncbi.nlm.nih.gov/pubmed/22750882

3. Bhat MA, Rios JC, Lu Y, Garcia-Fresco GP, Ching W, St Martin M, et al. Axon-glia interactions and the domain organization of myelinated axons requires neurexin IV/Caspr/Paranodin. Neuron. 2001 May;30(2):369–83.

4. Lux A, Yu X, Scanlan CN, Nimmerjahn F. Impact of Immune Complex Size and Glycosylation on IgG Binding to Human FcγRs. The Journal of Immunology. 2013;190(8):4315–23.

5. Wieske L, Martín-Aguilar L, Fehmi J, Lleixà C, Koel-Simmelink MJA, Chatterjee M, et al. Serum Contactin-1 in CIDP A Cross-Sectional Study Class of Evidence Criteria for rating therapeutic and diagnostic studies. Neurol Neuroimmunol Neuroinflamm. 2021;8.

6. Canetti D, Rendell NB, Gilbertson JA, Botcher N, Nocerino P, Blanco A, et al. Diagnostic amyloid proteomics: Experience of the UK National Amyloidosis Centre. Clin Chem Lab Med [Internet]. 2020 Jun 1 [cited 2020 Sep 15];58(6):948–57. Available from: https://www.degruyter.com/view/journals/cclm/58/6/article-p948.xml
