# Supplementary material for: Contactin-1 links autoimmune neuropathy and membranous glomerulonephritis
Source: PLoS One. 2023 Mar 9;18(3):e0281156. doi: 10.1371/journal.pone.0281156 (PMC9997925; doi:10.1371/journal.pone.0281156)
Supplement: S1 Raw images — (PDF) [file pone.0281156.s011.pdf]

Western blot uncropped unedited images

All blot, except 7C blot 3 (Odyssey XF LI-COR) images acquired through development using X-ray film and scanned to digitalise.

Figure 2C

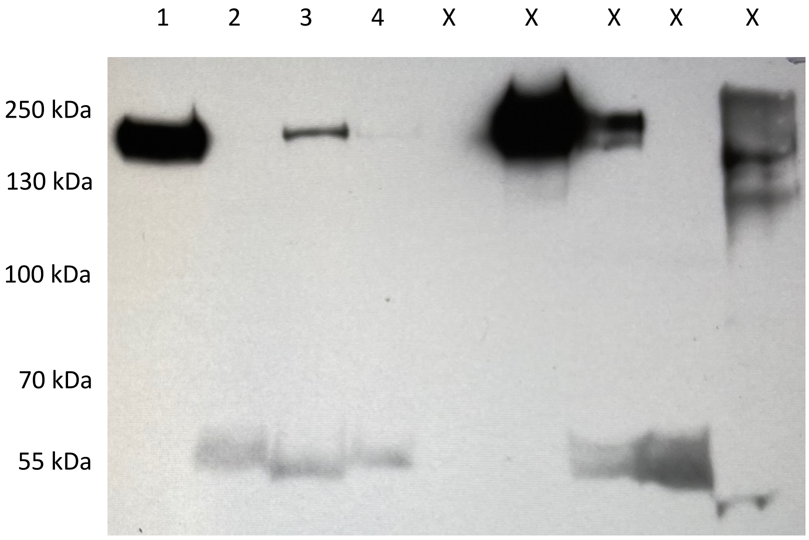

Lanes in order	Protein/Lysate loaded
1	Contactin-1 recombinant protein 0.003μg
2	Normal Human Serum (Immune complex precipitate)
3	P15 active disease (Immune complex precipitate)
4	P15 remission sera (Immune complex precipitate)
X	blank
X	Contactin-1 recombinant protein 0.01μg
X	Normal Human Serum (supernatant)
X	P15 active disease (supernatant)
X	P15 remission sera (supernatant)

Figure 2C (top blot lanes 5-8) and Supplementary figure 8 (S8)

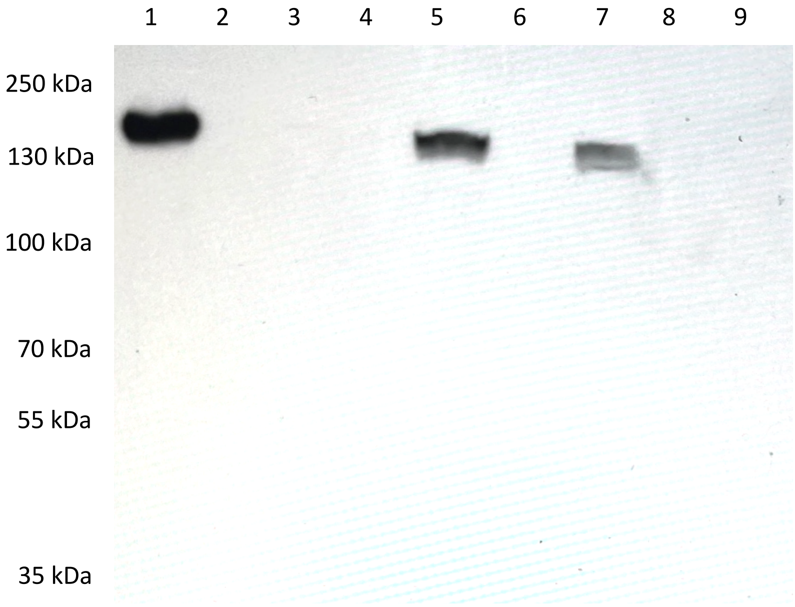

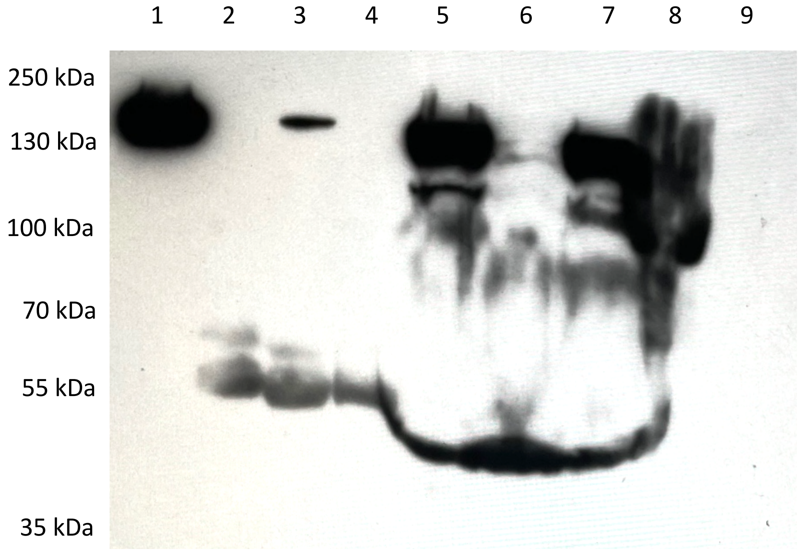

Both blots

Lane	Protein/Lysate loaded
1	Contactin-1 recombinant protein 0.01μg
2	Normal Human Serum (Immune complex precipitate)
3	P15 active disease (Immune complex precipitate)
4	P15 remission sera (Immune complex precipitate)
5	Normal Human Serum (supernatant)
6	P15 active disease (supernatant)
7	P15 remission sera (supernatant)
8	P15 active disease (whole sera)
9	Polyethylene glycol (blank)

Supplementary figure 7C (S7C)

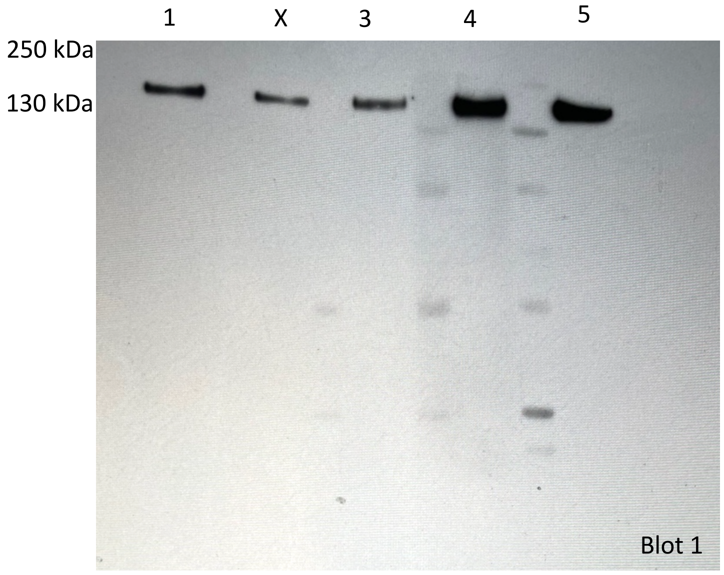

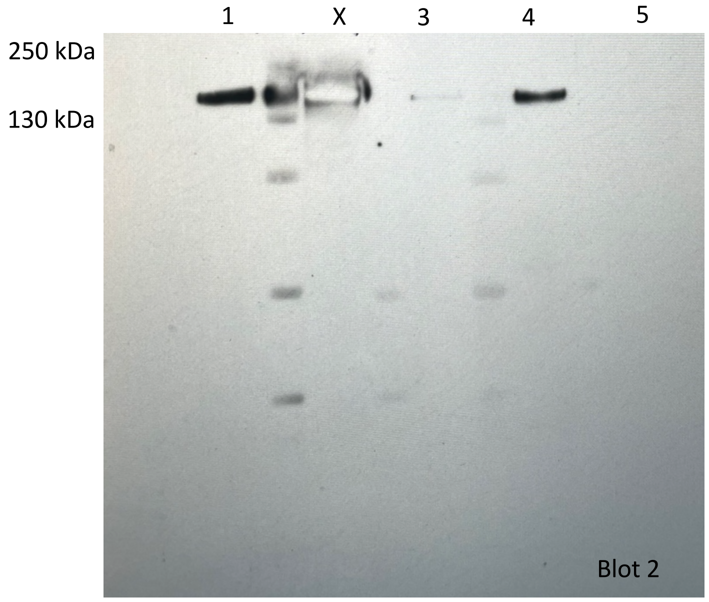

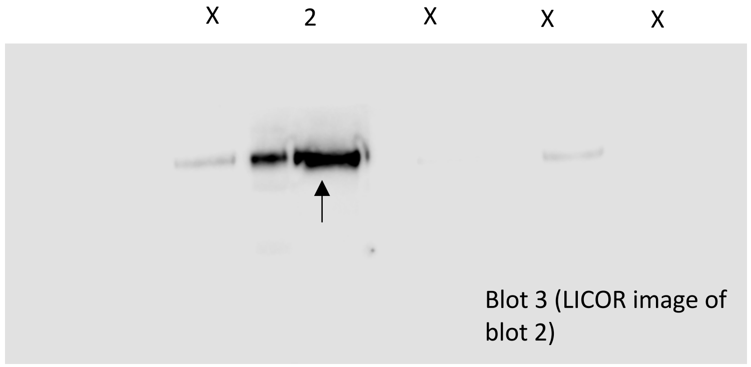

Note: figure is configured using both blots performed in parallel. Third image (blot 3) shows 'P12' from figure using blot 2 but imaged on as LICOR for optimal appearances (arrow).

Blot 1 (Gel loaded with recombinant protein and probed with following antibody or sera)

Lane	Protein/Lysate loaded
1	Goat anti-contactin-1
X	Goat anti-contactin-1
3	Patient 1
4	Patient 2
5	Patient 3

Blot 2

Lane	Protein/Lysate loaded
1	Patient 4
X	Patient 12
3	Patient 14
4	Patient 15
5	Pre-adsorbed Patient 16

Blot 3

Lane	Protein/Lysate loaded
X	Patient 4
2	Patient 12
X	Patient 14
X	Patient 15
X	Pre-adsorbed Patient 16
